# Predictive Model Construction for Social–Emotional Competence of Toddlers in Shanghai, China: A Population-Based Study

**DOI:** 10.3389/fpubh.2021.797632

**Published:** 2022-01-31

**Authors:** Deng Chen, Yilu Huang, Sikun Chen, Yunzhe Huang, Andrew Swain, Jinming Yu

**Affiliations:** ^1^Key Laboratory of Public Health Safety, Institute of Clinical Epidemiology, Ministry of Education, School of Public Health, Fudan University, Shanghai, China; ^2^Shanghai VIP Health Care Co., Ltd (C-LAP), Shanghai, China

**Keywords:** social-emotional development, toddlers, model construction, model evaluation, population-based study

## Abstract

**Objective:**

To construct a simple model containing predictors derived from Chinese Learning Accomplishment Profile (C-LAP) to better the evaluation of the social–emotional development of toddlers aged 24–36 months.

**Method:**

The test results by C-LAP system and demographic information of toddlers aged 24–36 months were collected between 2013 and 2019 in Shanghai, China, whose guardians were voluntary to accept the investigation. We developed a norm with the dataset based on the study population. With the norm, stepwise regression and best subset analysis were applied to select predictors.

**Results:**

Relying on the norm established and stepwise regression and also the best subset analysis, an optimal model containing only 6 indicators was finally determined and the nomogram of the model was constructed. In the training and validation dataset, the AUCs of the optimal model were 0.95 (95% CI: 0.94–0.96) and 0.88 (95% CI: 0.85–0.90), respectively. When the cutoff point of the model was set at 0.04, its sensitivity in training and validation dataset was 0.969 and 0.949, respectively, and the specificity in training and validation dataset is 0.802 and 0.736, respectively.

**Conclusion:**

A simplified predictive model which includes only 6 items derived from C-LAP is developed to evaluate the probabilities of being at risk of developmental problem in social–emotional development for toddlers aged 24–36 months. Meanwhile, specificity and sensitivity of the model may be high enough for future fast screening.

## Introduction

Social emotional competence is regarded as an important ability during the development of infants and toddlers, which includes the abilities to monitor and express both their negative or positive emotions, develop a kind relationship with peers and adults, and also explore and learn from their surroundings (1). Emde (2) and Stern (3) emphasized the importance of exchanges of affective information with social partners early in life and indicate that the affective exchanges that occur early in the development lay the foundation for more complex types of communication. It suggests that most toddlers aged 24–36 months old with typical development are able to show affection for friends without prompting and cooperate with other children, and they become more independent and more interested in new experience. Delays in social emotional development are considered to be associated with the increasing risk of some unpleasant outcomes at older age, comprising poor performance at school, mental problems, such as autism spectrum disorder, etc., (4, 5). According to a research that relies heavily on the questionnaires reported by teachers, 40% of the toddlers under 5 years more or less have the social–emotional vulnerabilities, including 8.8% of toddlers who have markably lower readiness to explore, 17.1% who demonstrated to have lower social skills, and approximately 9% of children who exhibited considerably higher aggression and hyperactivity than the others and those who had covulnerabilities (6). Promisingly, some preventions and interventions can be used to treat the infants and toddlers who are at high risk to get rid of the negative outcomes (7). The head start REDI program is just designed to promote academic and social–emotional school readiness as well as social behaviors (8, 9). In other words, early identification really matters in coping with such circumstance. Therefore, it is imperative to test the young children who may have social emotional developmental problems and refer them to professional institutions for further diagnosis.

To date, there have been numerous norm referenced instruments available used for assessing the social emotional competence of toddlers, such as the Infant-Toddler Social and Emotional Assessment (ITSEA), the Ages and Stages Questionnaire-3 (ASQ-3), Brief Infant-Toddler Social and Emotional Assessment (BITSEA), etc., (10–12). It is reported that these scales make the best of the abundant knowledge parents know about their children and have high reliability and validity (1). However, most of them can just give a single score or a group of scores, which are normative assessment comparing the result with normal children and they can not tell the parents or teachers what specific skill in social emotion the toddlers are deficient in. Fortunately, the criterion referenced tool can provide the information which the normative assessment lacks, and it is crucial to organically combine norm referenced and criterion referenced instruments to better evaluate the development of social emotion of toddlers (13, 14). The scale associated with social emotion contained in the Early Learning Accomplishment Profile (E-LAP) assessment system may meet the demand, which is a criterion-referenced assessment system developed in 1969, which is derived from many other famous classic scales. Incoporating items in sequential order, the E-LAP provides unique advantages over other more normative assessment tools (15). The E-LAP is designed to assist teachers, clinicians, and parents in assessing individual skill development in the six domains containing gross motor, fine motor, cognitive, language, self-help, and social emotion for children aged 0–36 months (15). In 2010, the E-LAP was introduced into China and currently has its Chinese version, Chinese Learning Accomplishment Profile (C-LAP), which has been proved to be of high reliability, validity, and responsiveness after its application in Shanghai, China (16).

But the items of the C-LAP are so numerous that the test needs quite a long time to be completed, which may not be so appropriate for routine screening use since a screening tool should be brief and adopted easily. Thus, given the importance of early identification of social emotional competence problems, in this paper we aim to develop a prediction model, derived from C-LAP, which can evaluate the social emotional development for toddlers aged 24–36 months. Moreover, this model is supposed to be as simple as possible to contain the most relative items. We also hope the model can help parents roughly know the child's development of social emotion, even if they have no access to test conducted by professional institutions.

## Methods

### Data Collection

**Figure 1:**
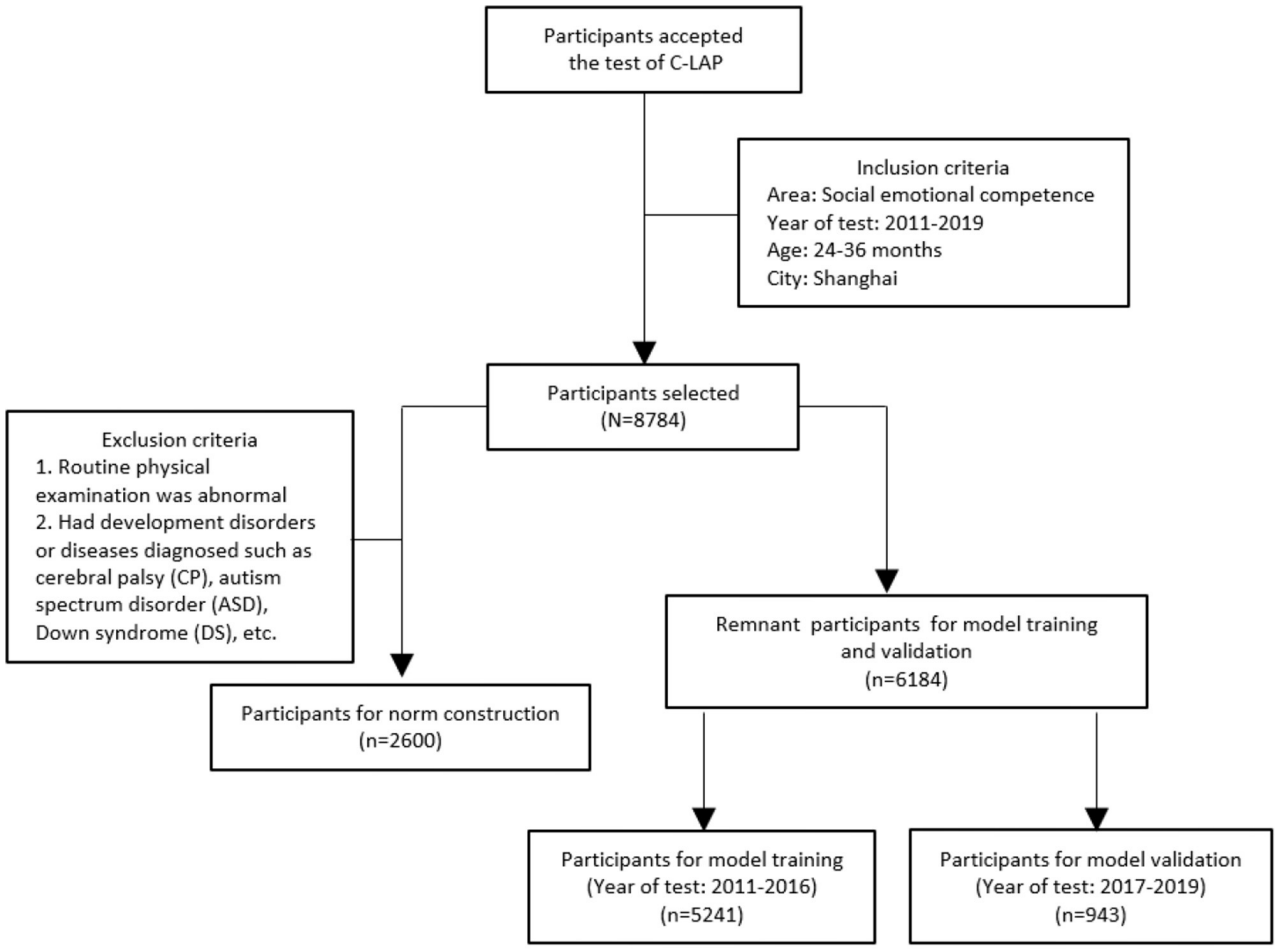
The flow diagram of participants selection and model construction. The flow chart showing the process of inclusion and exclusion of participants and the process of model construction.

When parents and their toddlers attended the selected Maternity and Child Care Centers and Community Health Centers in Shanghai, China for physical examination, the parents were approached by the staff member with a letter inviting their children to participate in this study. A total of 8,784 participants aged 24–36 months from Shanghai between 2011 and 2019 were recruited. Participants for norm construction needed to meet the following additional inclusion criteria: (a) routine physical examination was normal; (b) without diagnosed developmental disorders or diseases such as cerebral palsy (CP), autism spectrum disorder (ASD), down syndrome (DS), etc. The workflow of participants selection is shown in Figure 1. Every child participating in the study was tested by C-LAP to assess the developmental age, which is a measure of a child's development. The demographic characteristics, such as parent's education, age, and child's gender and chronological age were obtained by a standardized questionnaire. Besides, informed consent was obtained from their parents. The authors had no access to information that could identify individual participants during or after data collection. The study was conducted according to the guidelines of the Declaration of Helsinki and approved by the Institutional Review Board of the School of Public Health, Fudan University (IRB00002408, FWA00002399; approval number IRB#2019-04-0741). The written informed consent to participate was obtained from the parent or legal guardian of the children

### Measurement and Score

The context of the instrument was arranged from easy to difficult, and only children who pass the former questions were able to answer the latter ones. The first item corresponding to the physiological month age was selected as the starting point of the test; if a child passed it, then a plus sign would be recorded and the next item would be tested, and if not, a minus sign would be recorded and the researcher went back 8 items from it and reached a new starting point, and so on. After the test, a ceiling score and a basal score were obtained. The ceiling score was the top score a child could attain on a test regardless of ability or depth of knowledge, and the basal score was the first item number of the set of 8 consecutive items the child could successfully complete prior to the ceiling score.

Steps to score the C-LAP: (1) counted the number of minuses between the basal score and ceiling score; (2) subtracted the number of minuses from the ceiling score; (3) the age range where that number fell was the child's developmental age.

### Norm Construction

Lambda-Mu-Sigma (LMS) method was introduced to create reference centile curves to identify subjects who may be at the risk of social emotional developmental problems. The need for centile curves, rather than a simple reference range, arises when the measurement is strongly dependent on some covariates, often age, so that the reference range changes with the covariates. In the LMS method, the distribution at each covariate value is summarized by three parameters, the Box-Cox power I, the mean *p*, and the coefficient of variation cr. These three parameters are constrained to change smoothly as the covariate changes, and can, like the centiles, be plotted against the covariate. Thus, one advantage of this method is that the three curves, L, M and S, completely summarize the measurement's distribution over the range of the covariate.

In this study, the cutoff value of the “at the risk of social emotional developmental problems” was set at deciles of developmental age of social emotion of each age month. Given the long duration of data collection from 2011 to 2019, the whole time period was seperated into three shorter segments as “2011–2013,” “2014–2016,” and “2017–2019,” to determine whether the deciles varied with time.

### Model Selection

Subjects for model contruction and validation were classified by the time of their test records as training dataset (between 2011 and 2016) and validation dataset (between 2017 and 2019). Based on the pre-established norms, all of them were identified as “at the risk of social emotional developmental problem” or “not at the risk of social emotional developmental problem,” which served as the dependent variable of the predictive model.

There were a total of 38 items in the item bank of social emotion evaluation of C-LAP system. Although the sequence numbers of test item start for children aged 24–36 months were confined to 23–38, the items with sequence number from 1 to 22 were entirely in the possibility of being tested due to the particular test procedure of C-LAP. Thus, all the 38 items were initially incorporated as independent variables into the models, and then stepwise regression analysis was adopted for preliminary item selection. Next, the best subset analysis was applied to select an optimal model based on the predicators obtained from stepwise regression analysis, and the variable number in the best subset was identified.

### Model Evaluation

Area under curve (AUC) was adopted as the evaluation method of model predictive power. In general, its value between 0.8 and 0.9 indicates an excellent discrimination ability of the model, and over 0.9 indicates an outstanding one. And predictive ability of the model was demonstrated by the distribution of the classification of subjects deriving from the norm across the output risk possibility range between 0 and 1.

## Results

### Demographic Characteristic of Subjects for Building the Norm

Referring to relevant literature (17), 200 eligible subjects were randomly selected in each month age group from the whole samples, and a total of 2,600 samples were eventually used for norm construction. Of them, 1,367 (52.6%) were boys, and 1,233 (47.4%) were girls. The mean of chronological and developmental age of them was 30.0 (SD: 3.74) and 29.1 (SD: 5.28) months old, respectively. Most of their parents were about 30 years old, whereas mean age of fathers (31.3 years old) was a little older than that of mothers (30 years old). Education distribution of both parents was very similar, in which undergraduate and junior college students accounted for the vast majority (about 80%) (Table 1).

**Table 1:** Demographic characteristic information of participants for model training and validation.

**Variable**	**Norm** **dataset** **(*n* = 2,600)**	**Training** **dataset** **(*n* = 5,241)**	**Validation** **dataset** **(*n* = 943)**	**Overall** **(*N* = 8,784)**
**Chronological age (m)**				
Mean (SD)	30.0 (3.74)	29.8 (3.82)	29.3 (3.93)	29.8 (3.82)
Median [Min, Max]	30.0 [24.0, 36.0]	30.0 [24.0, 36.0]	29.0 [24.0, 36.0]	30.0 [24.0, 36.0]
**Developmental age (m)**				
Mean (SD)	29.1 (5.28)	29.1 (5.31)	28.2 (5.58)	29.0 (5.34)
Median [Min, Max]	28.0 [16.0, 36.0]	28.0 [12.0, 36.0]	28.0 [16.0, 36.0]	28.0 [12.0, 36.0]
**Gender**				
Male	1,367 (52.6%)	2,837 (54.1%)	496 (52.6%)	4,700 (53.5%)
Female	1,233 (47.4%)	2,404 (45.9%)	447 (47.4%)	4,084 (46.5%)
**Father's age (y)**				
Mean (SD)	31.3 (6.36)	30.8 (6.69)	33.5 (5.97)	31.2 (6.57)
Median [Min, Max]	31.0 [18, 55.0]	31.0 [18.0, 55.0]	33.0 [18.0, 51.0]	31.0 [18, 55]
**Mother's age (y)**				
Mean (SD)	30.0 (5.69)	29.5 (6.10)	32.2 (4.73)	29.9 (5.90)
Median [Min, Max]	30.0 [18, 45.0]	30.0 [18.0, 47.0]	32.0 [18.0, 42.0]	30.0 [18.0, 47.0]
**Father's education**				
High school and below	152 (5.8%)	898 (17.1%)	42 (4.5%)	1,092 (12.4%)
Associate degree	1,180 (45.4%)	1,842 (35.1%)	210 (22.3%)	3,232 (36.8%)
Undergraduate	876 (33.7%)	2,236 (42.7%)	586 (62.1%)	3,698 (42.1%)
Postgraduate	392 (15.1%)	265 (5.1%)	105 (11.1%)	762 (8.7%)
**Mother's education**				
High school and below	105 (4.0%)	944 (18.0%)	52 (5.5%)	1,101 (12.5%)
Associate degree	1,188 (45.7%)	1,879 (35.9%)	201 (21.3%)	3,268 (37.2%)
Undergraduate	913 (35.1%)	2,250 (42.9%)	608 (64.5%)	3,771 (42.9%)
Postgraduate	394 (15.2%)	168 (3.2%)	82 (8.7%)	644 (7.3%)

### Demographic Characteristic of Subjects for Model Training and Validation

Indicated by Table 1, for training dataset and validation dataset, the demographic characteristics were quite similar. The overall mean age of chronological age was about 29.7 (SD: 3.84) months, whereas the developmental age was around 29.0 (SD: 5.36) months. Their mother's mean age was 29.9 (SD: 5.98) years old, which was a little higher than their father's (31.2 ± 6.66 years old). Nearly half of their parents had received undergraduate degree.

### Norm

**Figure 2:**
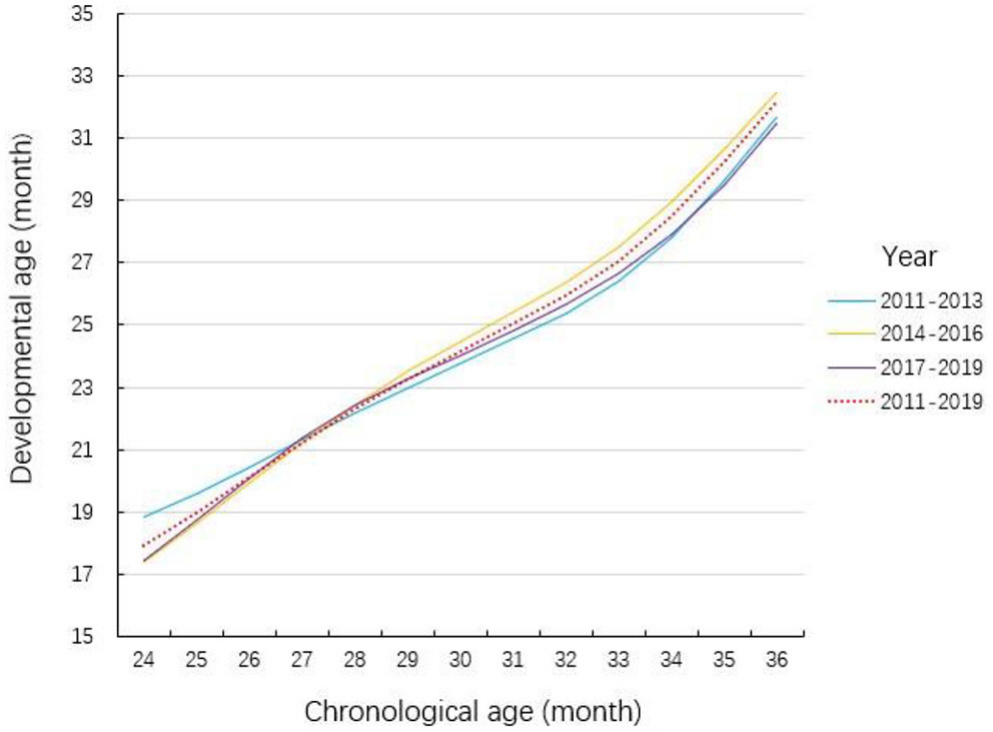
Comparison of the deciles of the social emotional development in different year intervals. The chronological age was calculated just based on the birthday of the participants while the developmental age was based on the result of C-LAP. The relationship between developmental age and chronological age is based on the dataset of 3 time period (2011–2013, 2014–2016, 2017–2019) as well as the whole dataset.

For every single chronological age month, through LSM, the developmental age month was determined to set at deciles in 2011–2013, 2014–2016, and 2017–2019. All these decile curves are shown in Figure 2, which indicated the deciles did not vary with time. Therefore, these 9 years' data were merged together, and the decile developmental age between 2011 and 2019 was calculated. Furthermore, for each age month, the difference between developmental age and chronological age was listed in Table 2, which was the standard for us in the following study to differentiate the participants who were at risk and who were not.

**Table 2:** Deciles (month) of social emotional development of children aged 24–36 months from Shanghai in different year intervals.

**Chronological age (Month)**	**Year 2011–2013** **(*n* = 864)**	**Year 2014–2016** **(*n* = 1,335)**	**Year 2017–2019** **(*n* = 401)**	**Year 2011–2019** **(*N* = 2,600)**	**Deciles of** developmental difference^*^
24	18.83	17.38	17.45	17.90	−6
25	19.61	18.65	18.75	18.98	−6
26	20.43	19.94	20.11	20.10	−6
27	21.31	21.22	21.39	21.23	−6
28	22.17	22.45	22.42	22.31	−6
29	22.98	23.52	23.26	23.27	−6
30	23.77	24.48	24.02	24.16	−6
31	24.57	25.41	24.81	25.03	−6
32	25.38	26.38	25.69	25.94	−6
33	26.40	27.52	26.68	27.04	−6
34	27.81	28.96	27.89	28.47	−6
35	29.64	30.65	29.51	30.24	−5
36	31.68	32.51	31.49	32.21	−4

* *indicates the developmental difference was obtained by subtracting chronological age from deciles of social emotional development in the year interval between 2011 and 2019*.

### Model Selection

**Figure 3:**
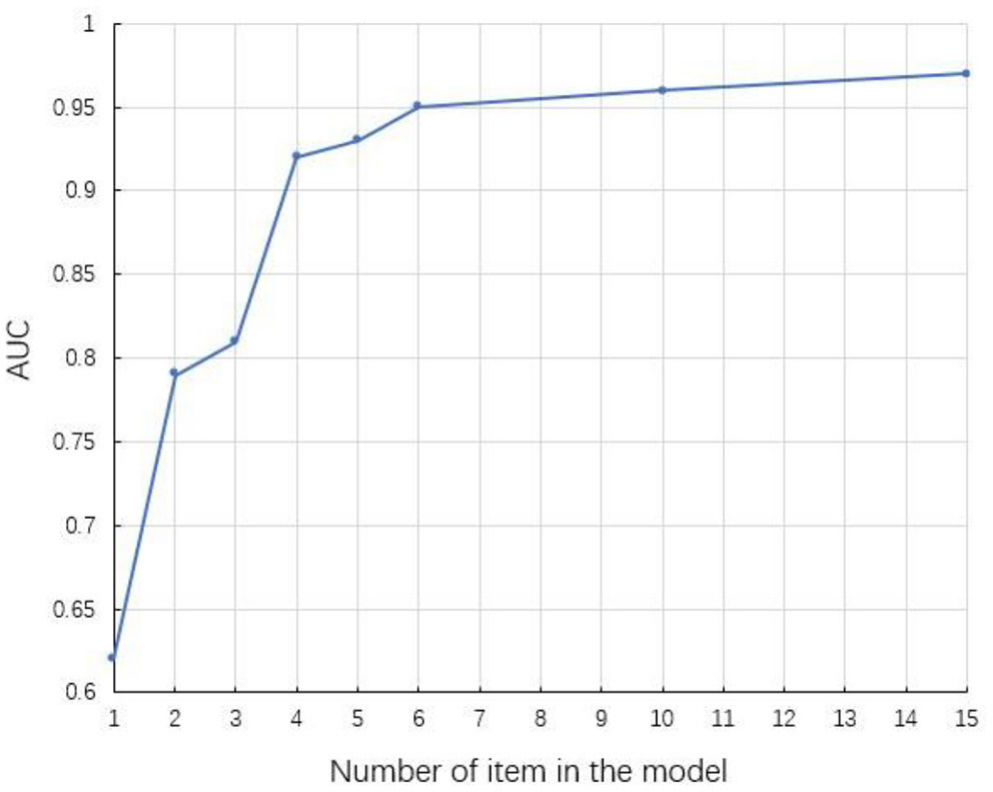
Area under curve (AUC) of the model in training dataset changes as the number of predictors increases. The predictors in the model include the chronological age.

**Figure 4:**
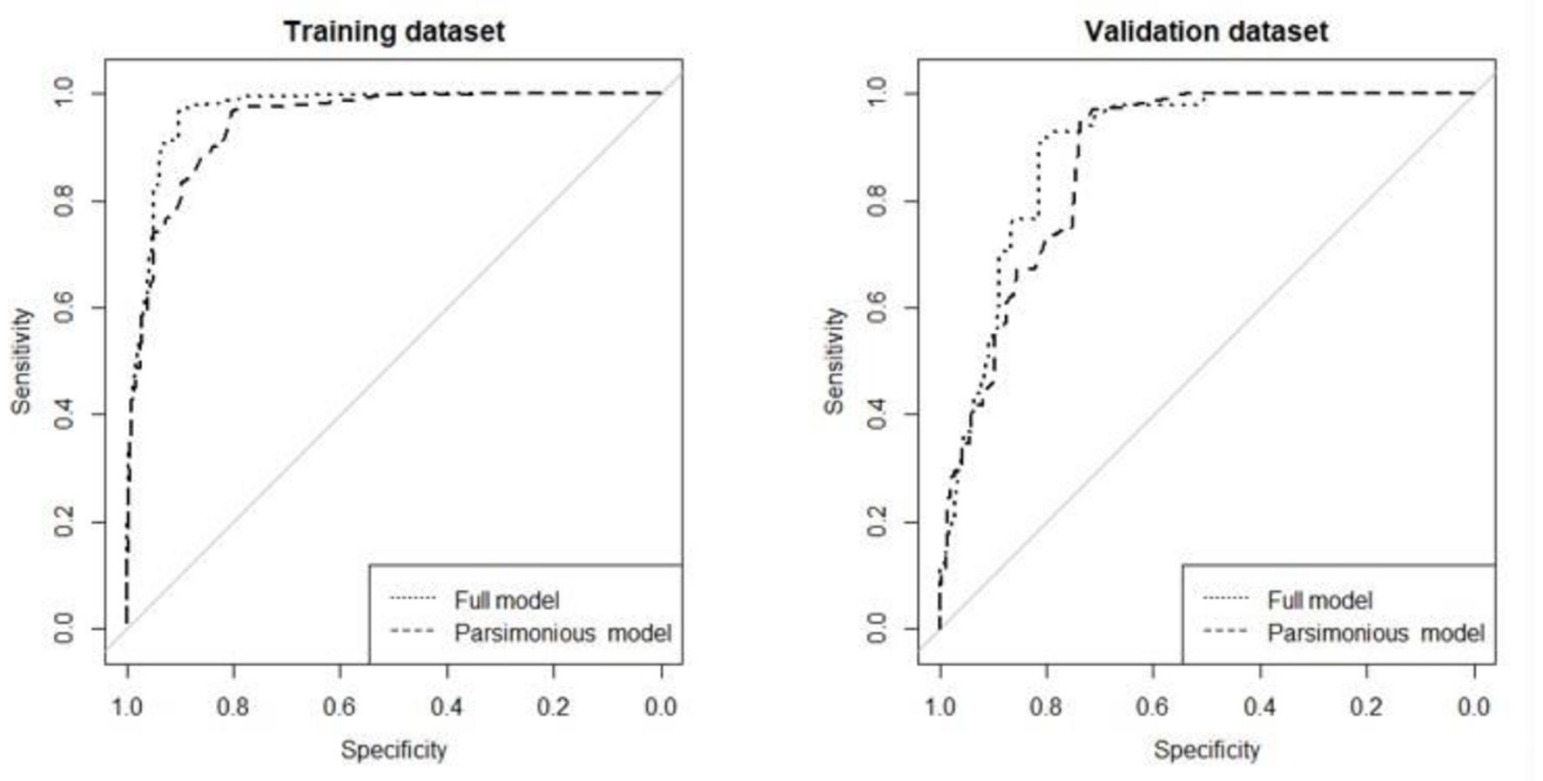
ROCs of full model and parsimonious model in the training dataset and validation dataset. Full model refers to the model that contains 15 predictors while the parsimonious model is the model that incorporates 6 predictors. In the training dataset, the AUCs of the full model and parsimonious model were 0.97 (95% CI: 0.96–0.98) and 0.95 (95% CI: 0.94–0.96), and in the validation dataset, the AUCs of the full model and the parsimonious model were 0.90 (95% CI: 0.87–0.92) and 0.88 (95% CI: 0.85–0.90), respectively.

Through stepwise regression, 14 items out of the whole 38 items and chronological age were extracted, which constitute the full model (Table 3). Then by applying best subset regression, the number of predictors included in the model could be further reduced, and balance the number of predictors with accuracy of model prediction. Figure 3 depicted the relationship between AUC and the number of predictors included in the model. It could be found that the AUC increases with the number of items contained in the model elevating. Besides, it was worth noting that, when the predictor number exceeded 6, the AUC increased much more slowly than before. Considering the “cost-efficiency” of the model, 6 predictors (chronological age and 5 items) were included in the parsimonious model (Table 3). ROCs of the full model and parsimonious model were provided in Figure 4. In the training and validation dataset, the AUCs of the full model were 0.97 (95% CI: 0.96–0.98) and 0.90 (95% CI: 0.87–0.92), whereas that of the parsimonious model were 0.95 (95% CI: 0.94–0.96) and 0.88 (95% CI: 0.85–0.90), respectively.

**Table 3:** Prediction model for the probability of “At risk of social emotional developmental problem” among children aged 24–36 months old in Shanghai, China.

**Full Model**						**Parsimonious Model**				
**Predictors**	**β**	**Odds Ratios**	**Std. Error**	**CI**	***P*-value**	**β**	**Odds Ratios**	**Std. Error**	**CI**	***P*-value**
(Intercept)	−0.56				0.785	−12.42				<0.001
Age	0.69	1.99	0.07	1.87–2.14	<0.001	0.55	1.74	0.05	1.64 – 1.84	<0.001
Item 20	−4.72	0.01	0.01	0.00–0.07	<0.001					
Item 21	−3.73	0.02	0.03	0.00–0.22	0.001					
Item 22	−2.19	0.11	0.09	0.02–0.55	0.006					
Item 23	−2.75	0.06	0.05	0.01–0.33	0.001					
Item 24	−1.82	0.16	0.09	0.05–0.50	0.002					
Item 26	−2.15	0.12	0.03	0.08–0.18	<0.001	−2.33	0.10	0.02	0.07 – 0.15	<0.001
Item 27	−1.6	0.2	0.04	0.13–0.31	<0.001	−2.03	0.13	0.03	0.09 – 0.20	<0.001
Item 29	−1.08	0.34	0.06	0.24–0.48	<0.001					
Item 30	−0.83	0.44	0.08	0.31–0.62	<0.001					
Item 31	−1.92	0.15	0.03	0.10–0.21	<0.001	−2.18	0.11	0.02	0.08 – 0.16	<0.001
Item 32	−1.96	0.14	0.03	0.10–0.20	<0.001	−2.18	0.11	0.02	0.08 – 0.16	<0.001
Item 33	−1.81	0.16	0.05	0.09–0.30	<0.001					
Item 34	−2.01	0.13	0.03	0.09–0.21	<0.001	−2.42	0.09	0.02	0.06 – 0.13	<0.001
Item 36	−2.38	0.09	0.05	0.03–0.24	<0.001					

*Item 20, “Toddlers give toys to adults as required;” Item 21, “Toddlers become more dependent on their mothers during the walking phase;” Item 22, “Toddler 's resistance to bedtime increases;” Item 23, “Toddlers want adults around;” Item 24, “Toddlers experience renewed anxiety about strangers;” Item 26, “Toddlers can pick up the toys and place them well according to the order;” Item 27, “Toddlers are able to play games with peers in a room;” Item 29, “Toddlers begin to calm down and defend their ownership of personal belongings;” Item 30, “Toddlers began to play by himself;” Item 31, “Toddlers enjoy role playing and they will wrap the doll tightly and put it on the bed;” Item 32, “Toddlers have routinized and compulsive-like movement;” Item 33, “Toddlers begin to play cooperative games;” Item 34, “Toddlers can name or and point to self in photograph;” Item 36, “Toddlers can recite nursery rhymes and songs”*.

### Model Evaluation

The Figure 4 showed AUC of parsimonious logistic regression model containing 6 predictors in the training dataset and the validation dataset.

**Figure 5:**
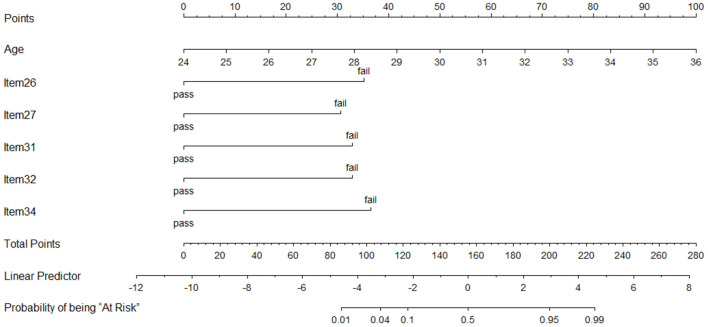
Nomogram of the parsimonious model. Points are assigned for age, item 26, item 27, item 31, item 32, and item 34 by drawing a line upward from the corresponding values to the “Points” line. The sum of these six points, plotted on the “Total points” line, corresponds to predictions of being at risk of developmental problem in social emotion. Steps to utilize this nomogram: first, draw a vertical line for each of the variables of a tested child (for example age = 27 months, item 26 = “pass,” item 27 = “fail,” item 31 = “pass,” item 32 = “fail,” item 34 = “pass”); then sum up the six values you read on the Points scale (25+0+30+0+32.5+0=87.5) to obtain the total points; finally draw a vertical line on the total points scale (87.5) to read the probability of being “At Risk” (between 0.01 and 0.04), and this indicates the child is not at risk of developmental problem in social emotion. Item 26, “Toddlers can pick up the toys and place them well according to the order;” Item 27, “Toddlers are able to play games with peers in a room;” Item 31, “Toddlers enjoy role playing and they will wrap the doll tightly and put it on the bed;” Item 32, “Toddlers have routinized and compulsive-like movement;” Item 34, “Toddlers can name or and point to self in photograph.”

**Figure 6:**
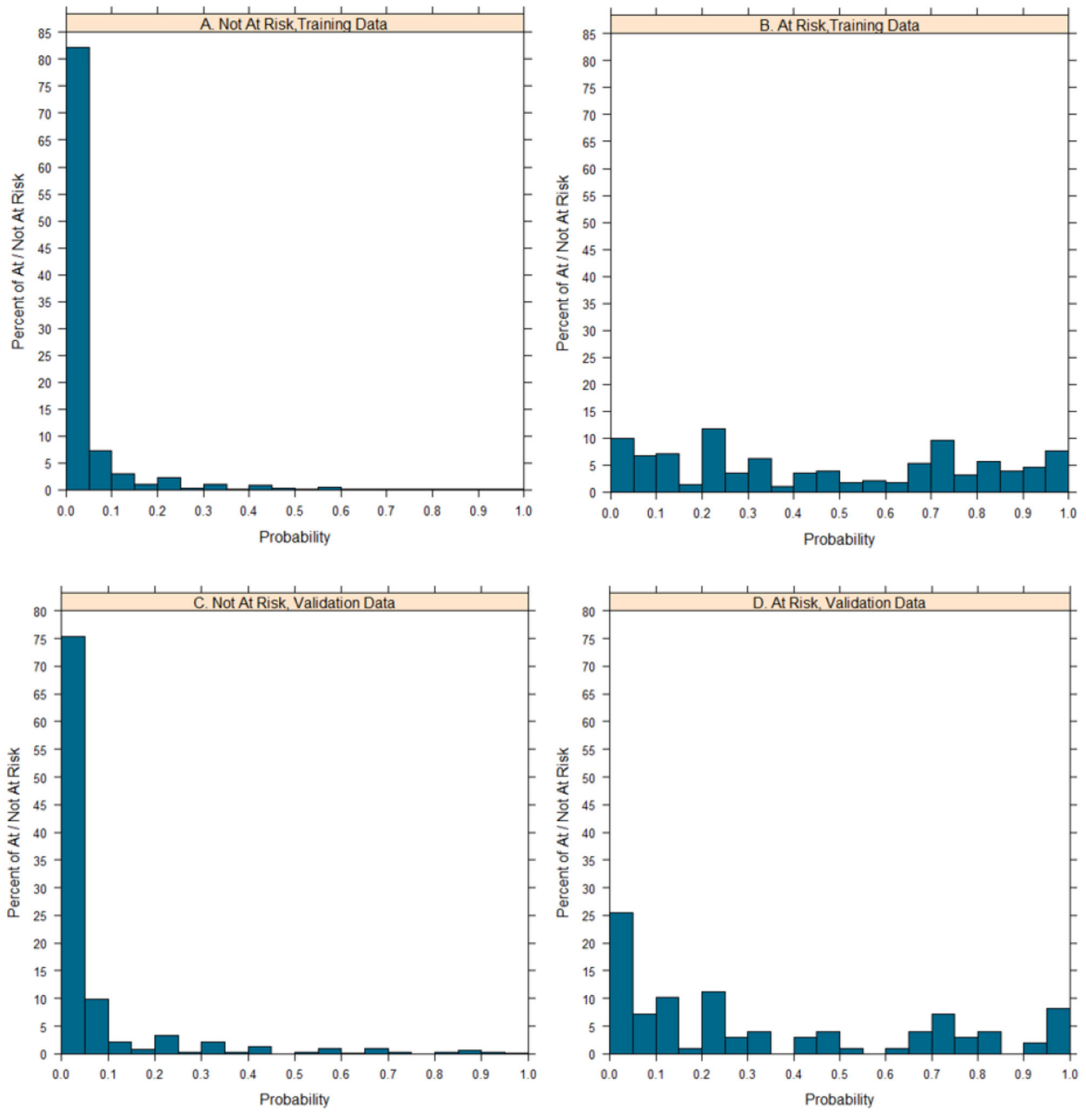
Distributions of probabilities of at/not at risk of developmental problem in social emotion of the parsimonious model. The distribution of at/not at risk of social emotional developmental problem probabilities, is shown among children who are at risk of social emotional developmental problem and who are not.

An equation was derived from the parsimonious model to calculate the probability of being at risk of developmental problem in social emotion [probability of being “At Risk” = e^x^/(1+e^x^)], X = −12.42 + (0.55 × Age) + (-2.33 × Item 26) + (-2.03 × Item 27) + (-2.18 × Item 31) + (-2.18 × Item 32) + (-2.42 × Item 34)). The nomogram of the model was constructed to provide accurately and visually individualized probability estimates of being “At Risk” (Figure 5). The nomogram assigned points based on age in a continuous and linear fashion. Points for items were assigned based on whether a child passed it. A calculated risk probability of 0.04 or higher of the model (nomogram) signifies the presence of “At Risk” and otherwise denotes that of “Not At Risk.” Given the cutoff value of 0.04, the sensitivity of training dataset was 0.969, whereas the specificity was 0.802. Similarly, the sensitivity of validation dataset was 0.949 and the specificity was 0.736. Furthermore, in the training dataset and validation dataset, model-predicted probabilities of risk of social emotional development problem discrimination showed good discrimination between participants who were judged as “At Risk” and who are “Not At Risk,” with only modest overlap (Figure 6).

## Discussion

A simplified predictive model with a nongram was constructed in this study containing 6 predictors, and the accuracy of the model is comparable to that of an instrument, BITSEA, a classical screener containing 42 items designed to identify children at risk for delays in social-emotional competence, the item selection of which is informed by clinical and empirical considerations (18) and the sensitivity and specificity of which are about 93 and 78%, respectively (10). The results in this study indicated that the model identified eventually is of high ability to identify toddlers who are at risk of delays in social–emotional competence.

In the model, the following predictors are mostly significant: “Toddlers are able to play games with peers in a room,” “Toddlers enjoy role playing and they will wrap the doll tightly and put it on the bed,” “Toddlers have routinized and compulsive-like movement. (e.g., they would like the specific person to take them to dine or they like the regular seat),” “Toddlers can pick up the toys and place them well according to the order,” “Toddlers can name or and point to self in photograph.” These items may work as different roles for evaluating the probabilities of being at risk of delay in social emotional development.

As is well-known, social fear might be a crucial factor associated with the toddler's state of social emotion. The indicator contained in the model “Toddlers are able to play with peers.” is to test whether the children have trouble getting along well with others due to social fear. Social fear refers to an emotion that describes anxiety, distress when toddlers are faced with totally new environment, with which, toddlers often behave less active and spend much time hesitating to play with peers(19). Generally speaking, fears occur at around 8 months old and then normally increase steadily as toddlers grow older and will finally stabilize at the second year (20). Although it is believed that moderate fear is an adaptive way to confront and explore the novel world, extreme fears will contribute to negative outcome associated with social emotion (21). Extreme social fears of toddlers are reported to directly and indirectly influence the interpersonal communication of toddlers (22). Some investigations indicated that children between 14 months and 7 years old with highly reported social fear was at higher risk of being diagnosed with social anxiety in adults (23). The special role of social fear in social development was implicated in the model when compared with other items in the original version of C-LAP.

“Toddlers enjoy role playing and they will wrap the doll tightly and put it on the bed.” This marks an ability to endow objects with imaginary functions and characteristics. Typically, children will spend much of their time in playing to explore their surroundings and by the second year after they were born, their behavior of playing will become more and more complicated and functional when they start to engage in pretend play in forms of acting directly to themselves or a doll or in a social manner, such as pretending to feed the doll as infants without real food (24). In some researches, the emergence of pretend play delays for toddlers at 24 months was identified as a very crucial marker of ASD, which was a poor outcome of social emotional development (25, 26). Consistent with the model, these researches highlighted the great significance of prentend play in the development of social emotion.

“Toddlers have routinized and compulsive-like movement” was also selected into the model, which was considered as a good indicator to well predict the risk of delay in the development in social emotion. However, the relationship between social emotional development and the behavior is actually not so clear. Some researches argued that such a behavior was prevalent among the toddlers between the ages of 8 and 72 months, which might indicate an adaptive function associated with cognition and social emotional development (21, 22, 27, 28). Although many other surveys claimed that young children's ritualistic behavior was more likely to be linked with fear, shyness, and failure of shifting emotion (23, 24, 29, 30), many others implicated that the behavioral differences between normal children and toddlers with mental problems were subtle (31). Therefore, whether the occurrence of the behavior alone is sensitive enough to identify children who are at risk of delay in social emotional development should be further determined in the future.

“Toddlers can pick up the toys and place them well according to the order.” This means the toddlers are able to understand the instructions and follow the verbal command. Generally speaking, it is an item that tests language and speech. Similarly, previous studies suggested that delays in language and speech referred to be associated with developmental disorders, which aligns with the results in this study, indicating the importance of the ability (32, 33).

“Toddlers can name or point to self in photograph” marks that children begin to have self-consciousness no matter how the surroundings change, which can be defined as an awareness of his or her body as well as its interactions with the environment and others. A review indicated that such an awareness did not arise until 2 years old and the development ended at the age of 3(34). Self-concept is often tested by the mirror test and a study found that most of the children in the middle of the 2nd year passed the test (35). It can be concluded that at around 24 months to 36 months, toddlers are supposed to have the ability of differentiating self from others (20). Neriser (36) described two unique ways to form self-awareness: one was having the body perception and the other was interacting with environment and people, which was also an essential part of social emotional competence. In other words, delay in differentiating self from others may be a result of something wrong with the social emotional development. Also, a review concluded that disturbances in self-image recognition could be considered as an indicator or a possible marker of psychological developmental problems (34). Therefore, the special relationship between social emotional development and the behavior is indicated by the results and the previous researches for children aged 24–36months. Also the item can serve as a good predictor to evaluate the probabilities of being at risk or not.

Chronological age was also included in the model. It may be reasonable that if two persons respond to the questions above in the same way, the elder may be at greater risk.

This study is based on a relatively large sample of toddlers aged 24–36 months old in Shanghai, which may increase the power of the model. Moreover, stepwise regression and best subset regression were combined to get the optimal model with high accuracy and efficiency. Furthermore, the data set collected in 2017–2019 was applied to test the fitness of the model. It turned out that the model was of a good external validity.

However, it can not be denied that this research does have some limitations. First, the data used for model construction and validation are retrospective and its accumulation process is not strictly controlled, which hinders us from obtaining detail sociodemographic information. Hence, the number of associated variables included currently is limited, and more associated information in future studies will be added. Second, all of the data were collected from toddlers whose parents volunteered to do the test. Bias may exist because the study population may not represent the general population. Luckily, the sample size may be large enough to compensate for it.

## Conclusions

In summary, a simple model with a nongram evaluating the social emotional development of toddlers was developed, which incorporated six items representing various related aspects by stepwise regression and best subset regression. In addition, specificity and sensitivity of the model were tested to be good enough for future application.

## Data Availability Statement

The raw data supporting the conclusions of this article will be made available by the authors, without undue reservation.

## Ethics Statement

The studies involving human participants were reviewed and approved by Institutional Review Board of the School of Public Health, Fudan University. Written informed consent to participate in this study was provided by the participants' legal guardian/next of kin.

## Author Contributions

AS and JY conceptualized the document. DC performed the data curation. DC, YiH, SC, and YuH had done investigation. DC, YiH, AS, and JY contributed to the methodology. DC and YiH were incharge of the software, wrote the original draft, and supervision. JY did writing-review and editing. All authors have read and agreed to the published version of the manuscript.

## Funding

JY is employed by DC. This study received funding from JY. The funder involved in the study design, decision to publish, and preparation of the manuscript.

## Conflict of Interest

Authors YuH and AS are employed by Shanghai VIP Health Care Co., Ltd (C-LAP), Shanghai, China. The remaining authors declare that the research was conducted in the absence of any commercial or financial relationships that could be construed as a potential conflict of interest.

## Publisher's Note

All claims expressed in this article are solely those of the authors and do not necessarily represent those of their affiliated organizations, or those of the publisher, the editors and the reviewers. Any product that may be evaluated in this article, or claim that may be made by its manufacturer, is not guaranteed or endorsed by the publisher.
